# Tailoring surface morphology on anatase TiO_2_ supported Au nanoclusters: implications for O_2_ activation†

**DOI:** 10.1039/d4na00744a

**Published:** 2024-09-18

**Authors:** Muhammed Fasil Puthiyaparambath, Julian Ezra Samuel, Raghu Chatanathodi

**Affiliations:** a Department of Physics, National Institute of Technology Calicut Calicut Kerala 673601 India raghuc@nitc.ac.in

## Abstract

Strong interaction between the support surface and metal clusters activates the adsorbed molecules at the metal cluster–support interface. Using plane-wave DFT calculations, we precisely model the interface between anatase TiO_2_ and small Au nanoclusters. Our study focusses on the adsorption and activation of oxygen molecules on anatase TiO_2_, considering the influence of oxygen vacancies and steps on the surface. We find that the plane (101) and the stepped (103) surfaces do not support O_2_ activation, but the presence of oxygen vacancies results in strong adsorption and O–O bond length elongation. Modifying the TiO_2_ surface with supported small Au_*n*_ nanoclusters (*n* = 3–5) also significantly enhances O_2_ adsorption and stretches the O–O bond. We observe that manipulating the cluster orientation through discrete rotations results in improved O_2_ adsorption and promotes charge transfer from the surface to the molecule. We propose that the orientation of the supported cluster may be manipulated by making the cluster adsorb at the step-edge of (103) TiO_2_. This results in activated O_2_ at the cluster–support interface, with a peroxide-range bond length and a low barrier for dissociation. Our modeling demonstrates a straightforward means of exploiting the interface morphology for O_2_ activation under low precious metal loading, which has important implications for electrocatalytic oxidation reactions and the rational design of supported catalysts.

## Abstract 2

Strong interaction between the support surface and metal cluster activates the adsorbed oxygen molecule at the metal cluster–support interface.

## Graphical abstract

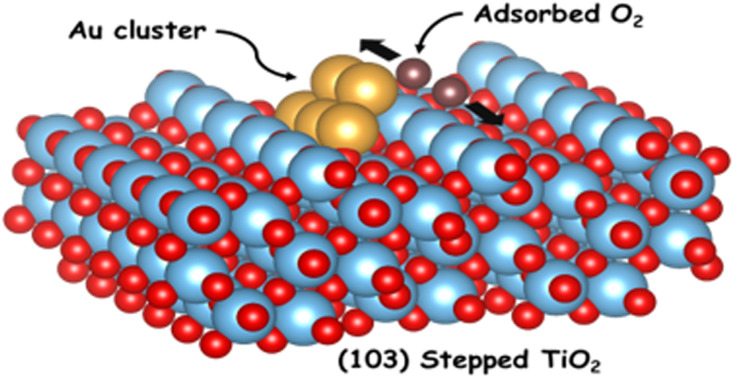

## 1. Introduction

Activation of molecular oxygen adsorbed over a catalyst surface is a critical step in several important processes such as catalytic oxidation reactions, photocatalysis, CO oxidation, propylene epoxidation, corrosion control, chemical sensing, *etc.*^1–4^ For example, the electrocatalytic oxygen reduction reaction (ORR) which occurs in hydrogen fuel cells is initiated by the adsorption and activation of an O_2_ molecule on a catalyst surface.^5^ The catalyst here may be a metal, non-metal or metal oxide. The progress of the ORR greatly depends on the bonding and activation of O_2_ on the active site on the catalyst surface. Understanding and controlling O_2_ activation provides a handle over phenomena like corrosion and oxide layer formation, which have immense practical implications.^6^ In general, the reactivity of a surface to O_2_ is indicated by the strength of the binding, measured in terms of the adsorption energy, the O–O bond length *etc.*^7,8^ Therefore, a study of how surface properties and their modification affect oxygen binding and activation can provide fruitful insights for desirable ends like controlling corrosion or designing efficient catalysts for oxidation reactions.

The study of O_2_ binding and activation on metal and alloy surfaces has received a fair bit of attention over the years, both experimentally and theoretically. A comprehensive review of the area may be found in the work of Montemore *et al.*^9^ With reference to the ORR in particular and electrocatalysis in general, the search for efficient and earth abundant catalysts to replace expensive and kinetically sluggish Pt or Pt-based materials is an ongoing research endeavour. Development of such alternative catalysts can lead to the large scale deployment of fuel cells in place of fossil fuels, which would lead to the realization of some of our green energy aspirations. One class of alternate materials being pursued here are metal oxides, particularly transition metal oxides. Transition metal oxides (TMOs) have gained significant interest over time as applicable active materials in the fields of catalysis, energy storage and sensing, due to their low cost, variable oxidation state, ease of synthesis, stability, corrosion resistance and environmental friendliness.^10–13^

Titanium dioxide (TiO_2_) is a very well-known material amongst TMOs, due to its relatively large abundance, non-toxicity and several applications as a wide band gap electronic material and a photocatalyst.^14^ TiO_2_ occurs in nature in the form of rutile and anatase, of which rutile is thermodynamically more stable, but anatase is catalytically and technologically an important TMO.^15^ Over the past decade or so, the study of interaction between TiO_2_ and O_2_ molecules has attracted considerable interest. It happens that the anatase TiO_2_ surface is relatively inactive to the O_2_ molecule, and therefore not a suitable catalyst for the ORR. The surface becomes active when an excess of electrons is present.^16^ The adsorption of O_2_ on TiO_2_ is generally associated with the withdrawal of negative charge from the surface.^17–19^ Adsorbed O_2_ exists in either a peroxide (O_2_^2−^), or superoxide (O_2_^−^) state, or may get dissociated into two O^2−^ ions.^9^

Varied attempts have been made to enhance the activity of the TiO_2_ surface towards O_2_. Adsorbed metal single atoms or nanoscale clusters, surface defects, steps and dopant atoms can play a significant role in activating the surface.^20^ The interface formed between the metal oxide surface and the metal clusters dispersed over it plays an important role in the catalytic oxidation processes.^21,22^ The geometric structure and electronic properties of the metal clusters can get significantly modified by the supporting metal oxides, and thereby have a significant impact on their catalytic properties.^23^ Gold nanoparticles exhibit high catalytic activity for many reactions when dispersed on metal oxides. Interestingly, both Au and metal oxides individually were considered to be inactive for numerous catalytic reactions.^24^ However, they combine to form a very active interface. The pioneering work of Haruta^25^ has led to extensive experimental studies on Au/oxide systems, yielding significant results, and it is established that the catalytic activity of Au-based systems greatly depends on the choice of the underlying oxides.

Among all the catalytic reactions involving Au, the activation of O_2_ holds particular significance in the research field of Au/oxide systems. Strategies are currently being developed for the activation of O_2_ molecules using the Au-metal oxide interface. It is found that the catalytic activity of Au depends considerably on the nature of the support, the synthesis method and the size of Au nanoparticles. The most commonly used oxide supports for Au nanoparticles are TiO_2_, ZnO, Al_2_O_3_ and SiO_2_.^26,27^ The structural aspects of Au clusters supported on TiO_2_ were investigated by Hemmingson *et al.* using high-resolution electron microscopy (HREM).^21^ It was discovered that the catalytic activity can be tuned by controlling the size of the Au nanoparticles, and the oxygen molecule is activated at the interface of Au/TiO_2_.^28–30^ Green *et al.*^31^ experimentally and theoretically provided insights into the activation of Au clusters supported on rutile TiO_2_. The transmission IR spectroscopic data indicated the direct involvement of the Au–Ti^4+^ at the interface in the activation of O_2_. While most researchers agree that the activation of O_2_ is favoured at the perimeter site, the elementary process leading to O_2_ activation, whether solely on the Au clusters or at the cluster–support interface, is debatable.^24,27,32,33^ Moreover, a decrease in the size of the clusters resulted in a decrease in the activity of Au clusters.^34^ The interactions between Au and TiO_2_ support have been extensively studied using density functional theory (DFT).^35,36^ The results suggest that the adsorption strength of Au/TiO_2_ is sensitive to both the cluster size and facet. Additionally, the interface's geometric and electronic structure plays crucial roles in the catalytic activity.

Stepped edges are common defects found on the surface of crystalline materials. Despite being ubiquitous, they have been studied less extensively compared to other defects such as vacancies and interstitials. High-index surfaces exhibit unique properties compared to low-index surfaces because of their low coordination.^37^ Surface facets characterized by Miller indices {*h*, *k*, *l*} with at least one index greater than one, can be termed stepped surfaces. The step edges serve as active sites for the adsorption of molecules and also act as nucleation centres when metals are deposited on the oxide surface.^38–40^ The stepped surface of anatase TiO_2_ is of great interest and has been amply investigated by Gong *et al.*^41,42^ They observed that, depending on the terrace/step configuration, the steps can exhibit lower reactivity compared to the flat terraces. Rieboldt^43^ and co-workers experimentally studied vicinal rutile TiO_2_ surfaces and found that they have a strong influence on O_2_ due to the presence of step edges. Additionally, these surfaces are characterized by smaller gap states compared to flat surfaces. High-resolution TEM images show that the (103) surface of anatase nanoparticle can be exposed depending on the processing conditions.^44^ The work function of the TiO_2_ (441) surface was smaller by 0.7 eV compared to (110)^45^ due to Fermi-level pinning and downward band bending toward the surface. Du *et al.* recently reported that the larger Au nanoparticles exhibit a higher encapsulation tendency in the Au/TiO_2_ system due to size-dependent strong metal–support interaction.^46^

In the present work, we have looked at O_2_ adsorption on plane, stepped, reduced and Au cluster decorated surfaces of anatase TiO_2_. We have used plane-wave DFT calculations to model O_2_ adsorption, probing adsorption energy and the O–O bond length as descriptors of O_2_ activation over these surfaces. We attempt to demonstrate that alteration of surface morphology of anatase TiO_2_ through the presence of vacancies and steps, as well as supported Au clusters, at minimal Au loading, can lead to enhanced O_2_ activation. We find that the orientation of the Au nanoclusters plays an important role in enhancing O_2_ binding to the surface. We point out a simple way to realize such optimal orientation, *viz.*, cluster adsorption at the step-edge in the stepped TiO_2_ surface. In the next section, we describe the computational methodology and in the subsequent section our results. Finally, we summarize and conclude our work.

## 2. Computational methods

All calculations in this work were performed using a spin-polarized density functional theory (DFT) approach with the Vienna *Ab initio* Simulation Package (VASP).^47,48^ The core electrons were treated using the projector augmented wave (PAW)^49,50^ method. The generalized gradient approximation (GGA) with the Perdew–Burke–Ernzerhof (PBE)^51^ functional was employed to describe the exchange–correlation energy. The DFT-D2 method proposed by Grimme^52,53^ was used to model the dispersive interactions. The wave functions were expanded in a plane wave basis with an energy cut-off of 520 eV, and Gaussian smearing of 0.05 eV was applied. The Brillouin zone was sampled using 5 × 5 × 1 Monkhorst–Pack grid^54^ for the surface slab. A 15 Å vacuum was included to avoid the spurious periodic interaction along the *z*-axis. The structural relaxation was carried out until the force on each atom was less than 0.01 eV Å^−1^. It is well known that the standard DFT fails to model Ti atoms, primarily due to the self-interaction error inherent in the functionals used in the GGA. The GGA+*U* method was employed to calculate the electronic structure to overcome this limitation. The value of Hubbard parameter *U* was set to 4.2 eV, which is similar to the previous work ^55,56^

The optimized lattice parameters for the bulk anatase TiO_2_ (*a* = 3.88 Å and *c* = 9.58 Å), and calculated bond lengths (Ti–O) in apical (2.01 Å) and basal (1.98 Å) directions agree with previous experimental values.^57^ In this work, we have considered the (101) plane and (103) stepped surfaces of anatase TiO_2_. The (101) and (103) surfaces were modelled using a 3 × 1 supercell consisting of 108 and 144 atoms, respectively. Au clusters were modelled in a unit cell of 15 × 15 × 15 Å, considering only the *Γ*-point. To identify the minimum energy path (MEP) for O_2_ dissociation on the TiO_2_ surface, the climbing image-nudged elastic band (CI-NEB)^58^ method was utilized to determine the transition state. The activation energy is defined as *E*_a_ = *E*_TS_ − *E*_IS_, where *E*_TS_ and *E*_IS_ are the energies of the transition state and initial state, respectively. Bader charge^59^ analysis was employed to determine the local charge of the atom.

The surface energy (*γ*) is calculated using the expression:1
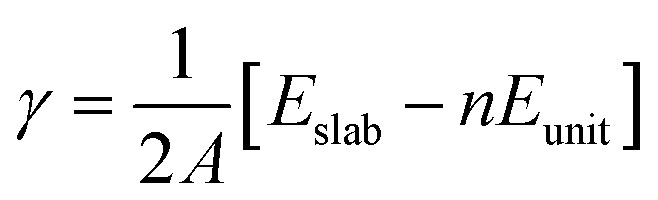
where *A* and *E*_slab_ represent the surface area and the total energy of the slabs, respectively, *E*_unit_ is the energy of TiO_2_ unit in bulk, and *n* stands for the number of the basic TiO_2_ units in the slab.

The adsorption energy of the molecular O_2_ is determined as:

2 *E*_O_2_ ad_ = *E*_O_2_+surf_ − *E*_surf_ − *E*_O_2__

where *E*_O_2_+surf_, *E*_surf_, and *E*_O_2__ denote the total energy of O_2_ adsorbed on the slab surface, the bare slab, and an isolated O_2_ molecule respectively. A more negative value indicates stronger adsorption. The usual gas phase O_2_ bond length is 1.23 Å. The strength of O_2_ adsorption can also be evaluated by the stretching of the O–O bond length, which is used as one of the descriptors for O_2_ adsorption.

The O vacancy is created in both (101) and (103) surfaces, with the formation energy given by the following equation:

3 *E*_f_ = *E*_def_ − *E*_pri_ − (1/2)*E*_O_2__

where *E*_def_ is the energy of the defective surface, *E*_pri_ is the energy of the pristine slab and *E*_O_2__ is the energy of the O_2_ gas molecule in its ground state.

The binding energy per Au for the Au_*n*_ cluster on the TiO_2_ substrate is calculated using the formula

4 *E*_BE_ = (1/*n*)[*E*_tot_ − *E*_slab_ − *n* × *E*_Au_]

where *E*_tot_ is the total energy of Au/TiO_2_, *E*_slab_ is the energy of the relaxed (101)/(103) slab, *E*_Au_ is the energy of an isolated single Au atom and *n* is the number of Au atoms on the slab. The negative value of *E*_BE_ indicates energetically favourable interaction between Au clusters and TiO_2_ surfaces. We have also performed *ab initio* MD (AIMD) simulations to study the stability of the Au clusters supported on TiO_2_ at room temperature.

## 3. Results and discussion

### 3.1 O_2_ adsorption on (101) and (103) anatase TiO_2_

**Fig. 1:**
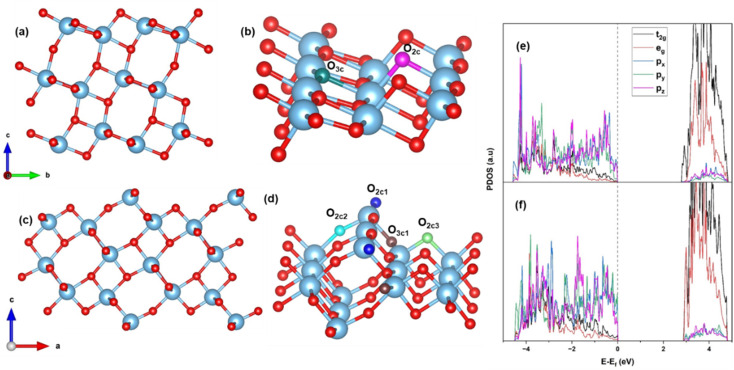
(101) TiO_2_ (a) side view (b) close view; (103) TiO_2_ (c) side view (d) close view; PDOS of (e) (101) and (f) (103) TiO_2_.

Bulk anatase TiO_2_ is cleaved into two different facets, the usual (101) and the stepped (103). The surface energy is calculated using the formula (1). The (101) facet has a surface energy of 0.46 J m^−2^ which is comparable to the previously calculated values.^60^ The optimized configuration of (101) is shown in Fig. 1(a) and (b). The (101) surface consists of fully saturated six-coordinated Ti and three-coordinated O atoms, as well as unsaturated five-coordinated Ti and two-coordinated O atoms on the surface. The (103) facet has two possible terminations that are commonly discussed: the “faceted” (103)_f_ and “smooth” denoted as (103)_s_.^60^ The surface energy of (103)_f_ is 1.12 J m^−2^, which is greater than 1.08 J m^−2^ of (103)_s_. This trend is similar to that observed in previous calculations,^61^ and this surface energy is lower than that of (001).^62^ Hence, the (103)_s_ is stable, and we consider the (103)_s_ facet throughout our calculation, denoted by (103). The optimized configuration is shown in Fig. 1(c) and (d). The hydration surface energy is also calculated for the (103) surface (for details, see S1†) at different coverage, and it is interesting to note that the surface energy of (103) is reduced for this coverage, when compared to those of (101). This indicates that hydration stabilizes the (103) surface over the (101).

The optimized configuration of the (103) surface consists of four-coordinated Ti (Ti_4c_) and three types of two-coordinated O (O_2c_), in addition to the usual coordination present in (101) anatase TiO_2_, and one three-coordinated (O_3c1_) (see Fig. 1(c) and (d)). The three types of O_2c_ are: (a) linear along the *b*-axis making an angle of 152° (O_2c1_), (b) inclined at an angle of 161° (O_2c2_) and (c) bend at an angle of 101° (O_2c3_). Relaxation of the (103) stepped surface leads to satisfaction of unsaturated surface bonds and a 3% stabilization of the system. In the relaxed geometry, the angle between Ti_4c_–O–Ti_4c_ along the *b*-axis is contracted by 4°, while the angle made in the *b*–*c* plane is increased by 5°.

To compare the electronic structure of two facets, the PDOS of (101) and (103) are calculated and plotted as Fig. 1(e) and (f), respectively. No significant change in the band gap is observable. In both cases, the top of the valence band (VB) is composed of O 2p states, and the bottom of the conduction band (CB) is composed of Ti 3d states, consistent with the previous literature.^63^ In (101), the valence band maximum (VBM) is dominated by p_*x*_ and p_*z*_, and the conduction band minimum (CBM) is composed of the dominant t_2g_ states of Ti (d_*xy*_, d_*yz*_, and d_*xz*_). In case (103), all the states of O 2p are present in the VBM, and the CBM consists of Ti 3d. This may be due to the lowering of the symmetry following the formation of a surface. The Ti–O–Ti bond angles of (103) are larger compared to (101), resulting in better availability of oxygen for bonding with other species. All these contribute to making the (103) stepped surface a more active site for binding of adsorbates.

However, we find that the binding of the O_2_ molecule to the (103) facet improves only marginally, despite all considerations of the previous paragraph. The adsorption energy of the O_2_ molecule is calculated on (101) and (103) using formula (2). The O_2_ molecule gets physisorbed in both cases. There is a slight increase in the adsorption energy of O_2_ in the case of the stepped surface (−0.17 eV) compared to the plane (−0.13 eV) surface of TiO_2_. The gas phase O–O bond length of 1.23 Å remains unaltered after adsorption. Thus, O_2_ molecules desorb easily from (101) and (103) TiO_2_ surfaces.

### 3.2 O_2_ adsorption on reduced (101) and (103) TiO_2_ surfaces

**Fig. 2:**
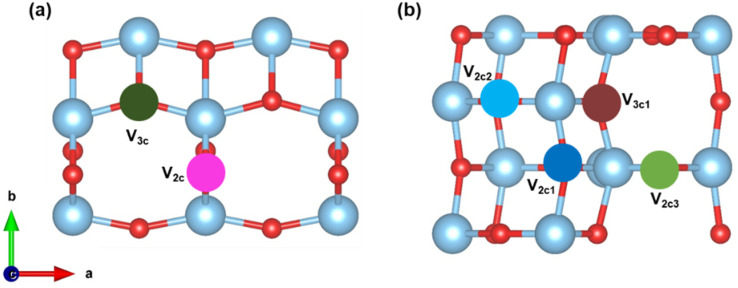
The top view of the vacant surface of (a) (101) and (b) (103).

We now discuss the case of oxygen defects on the TiO_2_ (101) and (103) surfaces. The formation energy of oxygen vacancies for both (101) and (103) surfaces is calculated using eqn (3) and is tabulated in Table 1. A large positive value indicates that more energy is needed to form the defect. The surface site defects V_2c_ and V_3c_ correspond to the vacancies created upon the removal of an O_2c_ and O_3c_ atom from the (101) surface (see Fig. 2(a)). Thus, it takes more energy to remove the O_3c_ than the O_2c_ atom. In the case of the (103) surface, V_2c1_, V_2c2_, V_2c3_, and V_3c1_ indicate the removal of O_2c1_, O_2c2_, O_2c3_ and O_3c1_ atoms, respectively, from the surface (see Fig. 2(b)). The formation energy is lowest for the removal of the O_2c3_ atom compared to all other sites.

**Table 1:** Computed formation energy values for defects on TiO_2_ (101) and (103) surfaces

Surface	Site	Formation energy (eV)
(101)	V_2c_	4.23
	V_3c_	5.02
(103)	V_2c1_	5.43
	V_2c2_	4.71
	V_2c3_	3.22
	V_3c1_	4.93

We create oxygen vacancies both on the plane (101) and stepped (103) TiO_2_ (shown in Fig. 2) and compare both cases. After relaxation, in the case of V_2c_ (removing the O_2c_ atom from the (101) surface), the Ti atom moves downwards by a distance of 0.16 Å, while the bottom oxygen atom is shifted upward by a distance of 0.18 Å. For the V_3c_ (removing the O_3c_ atom from the (101) surface) vacancy, there was no change in the position of the Ti atom in the *c*-direction, but it is shifted by 0.18 Å in the lateral direction. On the vacant surface with V_2c1_, the uncoordinated Ti atom was shifted away from the vacant site by 0.42 Å. Consequently, the adjacent Ti–O bond distance was reduced by 0.20 Å. On the V_2c2_ vacant surface, the Ti has been displaced to the vacant site by a distance of 0.40 Å. At the V_2c3_ vacant site, the bottom oxygen atom shifted upwards by 1.08 Å, while the six-coordinated Ti atom moved along the surface by 0.50 Å. On the V_3c1_ vacant surface, the oxygen atom originally coordinated to O_2c1_ becomes three-coordinated, leading to a reduction in the Ti–O bond distance from 2.46 Å to 2.11 Å. These geometries are displayed in Fig. S2.†

The adsorption energy of an O_2_ molecule is calculated on the reduced surfaces of (101) and (103). The adsorbed O_2_ configuration is shown in Fig. S3.† In the case of V_2c_ and V_3c_ of (101), the O_2_ molecule adsorbs at the Ti site, with O–O stretching of 1.46 Å and 1.48 Å, respectively, bringing these to the peroxide regime. The adsorption energy, O–O bond length and charge transfer to the molecule are tabulated in Table 2. The capability to absorb more than one oxygen molecule and activate these is important to enhance the efficiency of processes like the ORR. The study of O_2_ coverage can provide insights into the correlation between excess electron charge and the reactivity of the TiO_2_ surface.^16^ For more than one O_2_ adsorbed, we report the average value of adsorption energy, bond length and charge transfer. As the adsorbed O_2_ acts as an electron scavenger, it inhibits further adsorption of O_2_ molecules. We observe that the average adsorption energy is lowered, in the case of two molecules, with reduced stretching of the O–O bond. The average charge transfer is also lower in the case of two O_2_. What happens is thus: the first O_2_ molecule becomes trapped at the lattice site upon adsorption, thereby withdrawing all available electron density and thus, the second molecule is only physisorbed on the nearest Ti site available. These optimized configurations for V_2c_ and V_3c_ are shown in Fig. S4.†

**Table 2:** Oxygen adsorption on the reduced (101) TiO_2_ surface

	No. of O_2_ molecules	Sites	
		V_2c_	V_3c_
*E*_ads_ (eV)	1	−3.70	−4.35
	2	−1.94	−2.24
*d*_O−O_ (Å)	1	1.46	1.48
	2	1.34	1.36
*Q* |*e*|	1	0.92	1.08
	2	0.46	0.54

The average adsorption energy, bond length and charge transferred for adsorption of up to three O_2_ on the reduced (103) surface are tabulated in Table 3. The first O_2_ is adsorbed strongly, with a bond length in the peroxide regime. This is comparable with the case of the reduced (101) surface. In the case of V_2c1_, the adsorption of the second O_2_ molecule is much reduced, while there is practically no binding for the third. On the other hand, for V_2c2_, V_2c3_ and V_3c1_ sites, the first adsorbed O_2_ does not fill the vacant lattice site. The configuration of this adsorbed O_2_ is displayed in ESI Fig. S4.† Therefore, the second O_2_ has significant average adsorption energy, on the nearest Ti site available. This adsorbed O_2_ has a bond length in the superoxide regime. The third O_2_ is weakly bound, by physisorption since there is hardly any charge available on the surface, following the second binding. Thus, we see a graded lowering of the average adsorption energy and bond length with the number of O_2_ adsorbed in Table 3.

**Table 3:** Oxygen adsorption on the reduced (103) TiO_2_ surface

	No. of O_2_ molecules	Sites			
		V_2c1_	V_2c2_	V_2c3_	V_3c1_
*E*_ads_ (eV)	1	−5.09	−3.76	−3.02	−2.73
	2	−2.68	−2.47	−1.97	−2.35
	3	—	−1.56	−1.52	−1.66
*d*_O−O_ (Å)	1	1.46	1.47	1.46	1.45
	2	1.34	1.33	1.33	1.34
	3	—	1.30	1.29	1.30
*Q* |*e*|	1	1.10	0.82	0.86	0.88
	2	0.56	0.50	0.48	0.52
	3	—	0.34	0.32	0.35

In order to understand further the capture of O_2_ by a vacant surface site, we performed CI-NEB calculations on reduced (101) and (103) TiO_2_ and the details of CI-NEB can be found in S2.† We found that an energy barrier exists for the adsorbed O_2_ to move to the vacant lattice site, in the case of V_2c2_, V_2c3_, and V_3c1_ on the reduced (103) surface. This barrier is estimated to be 1.15 eV, 1.98 eV, and 0.78 eV respectively (see Fig. S5†). This is not the case for V_2c1_ on reduced (103), or V_2c_ and V_3c_ on reduced (101), where the transfer is barrierless. It may hence be concluded that the advantage of vacancies on reduced (103) TiO_2_ is that these provide multiple sites for O_2_ activation, hosting more than one activated O_2_ (as seen from Fig. S4†), resulting in improved efficiency for catalytic reactions.

### 3.3 O_2_ adsorption over Au clusters supported on (101) and (103) TiO_2_

Next, we investigate a strategy to enhance O_2_ activation over low loading of small Au clusters supported on (101) and (103) TiO_2_ surfaces. Small Au clusters have been rigorously studied over several years, owing to a wide range of applications in catalysis, nanophotonics *etc.*^64^ The formation of energetically stable Au_*n*_ on the (101) surface is discussed by Wan *et al.*^35^ and Vittadini *et al.*^65^ To find a stable structure for Au_*n*_, (*n* = 3–5), on stoichiometric (101) and (103), we grow the cluster over the surface step-by-step, adding one Au atom at a time and re-optimizing the geometry of the entire system. In this case, the binding energy per Au is calculated using eqn (4).

**Fig. 3:**
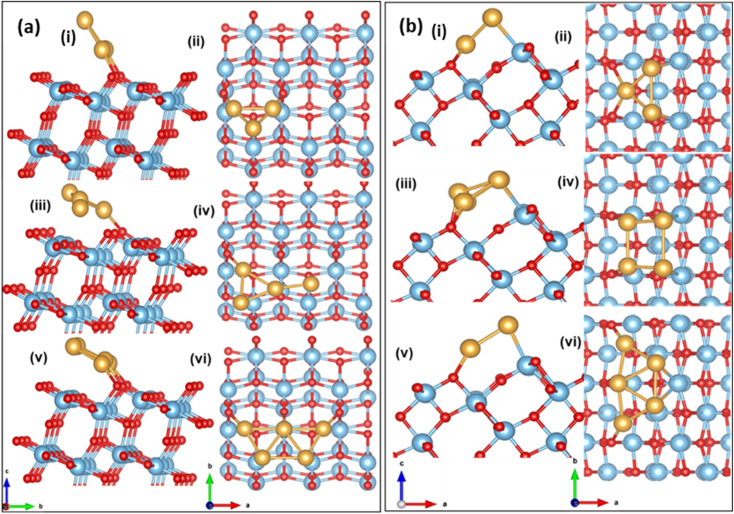
Side view and top view of the optimized configuration of Au_*n*_/TiO_2_ (a) (101); (b) (103) (i and ii) *n* = 3, (iii and iv) *n* = 4, (v and vi) *n* = 5.

The optimized configuration of an isolated Au_3_ cluster exhibits a planar, triangular geometry, which is more stable than the linear trimer. After adsorption and relaxation, the two Au atoms of the Au_3_ cluster bind to the O_2c_ of the (101) TiO_2_ surface with a binding energy of −2.28 eV and an Au–O_2c_ bond length of 2.04 Å. The Au_3_ cluster is inclined at an angle of 120°, to the *a*-axis. The optimized configuration is shown in Fig. 3(a) (i and ii). In Au_4_/TiO_2_, the fourth Au atom is attached to the optimized Au_3_/TiO_2_ structure and has a binding energy of −2.37 eV. Of the two Au atoms attached to the O_2c_ in the case of Au_3_/TiO_2_, one Au gets detached in the case of Au_4_/TiO_2_ and is tilted at an angle of 148°. The optimized configuration is shown in Fig. 3(a) (iii and iv). The most stable Au_5_ on (101) TiO_2_ is in the form of a planar trapezoidal structure. The three Au atoms are bonded with O_2c_ with an Au–O_2c_ bond length of 2.13 Å with a binding energy of −2.50 eV. The Au_5_ cluster gets tilted at an angle of 154°. The stable structure is shown in Fig. 3(a) (v and vi).

Au_*n*_ (*n* = 3–5) has different possible binding sites on the (103) surface. Among these, three types of two-coordinated oxygen sites (O_2c1_, O_2c2_, and O_2c3_) are present. The planar Au_3_ cluster is grown atom by atom, and the binding energy is calculated using eqn (4). The favourable configuration of Au_3_ has a binding energy of −2.39 eV at the O_2c2_ site with an Au–O_2c2_ bond length of 2.24 Å. The optimized configuration is shown in Fig. 3(b) (i and ii). The Au_3_ cluster on (103) is tilted at an angle of 48° to the *b*-axis. The Au_4_ cluster binds to the (103) with a binding energy of −2.62 eV and adopts a square geometry after adsorption. The bond lengths Au–O_2c2_ and Au–Ti are 2.08 Å and 2.72 Å, respectively. The cluster is tilted at an angle of 38° (see Fig. 3(b) (iii and iv)). The Au_5_ cluster binds on the (103) surface in a trapezoidal geometry with a binding energy of −2.70 eV and has an Au–O_2c2_ bond length of 2.20 Å, with a tilting angle of 40° (see Fig. 3(b) (v and vi)). The binding energy of the cluster in case of stepped surfaces is higher due to the availability of more states at the step edge.

The adsorption of O_2_ molecules on the optimized configuration of Au supported (101) and (103) surfaces has been investigated, and the results are tabulated in Table 4. Here, we have considered O_2_ adsorption on Ti atoms at the interface and low-coordinated Au sites on the cluster. At the interface, the preferred adsorption sites are Ti_5c_ and Ti_4c_, for (101) and (103) respectively. As Table 4 shows (see col. 4 and 8), there is weaker adsorption for O_2_ on the Au cluster itself, which is significant only in the case of the (103) surface.

**Table 4:** The adsorption energy (in eV) and the O–O bond length (in Å) for the O_2_ molecule on Au_*n*_ (*n* = 3, 4 and 5) supported on (101) and (103) surfaces, considering the active sites on Ti_5c_ (101)/Ti_4c_ (103) and Au

Au_*n*_ (*n*)	(101)				(103)			
	*E*_ads_ (eV) (Ti_5c_)	*d*_O−O_ (Å)	*E*_ads_ (eV) (on Au)	*d*_O−O_ (Å)	*E*_ads_ (eV) (Ti_4c_)	*d*_O−O_ (Å)	*E*_ads_ (eV) (on Au)	*d*_O−O_ (Å)
3	−0.73	1.32	−0.39	1.27	−1.66	1.43	−0.78	1.30
4	−0.44	1.32	−0.38	1.24	−1.88	1.44	−0.90	1.36
5	−0.99	1.34	−0.85	1.32	−1.84	1.44	−1.10	1.35

**Fig. 4:**
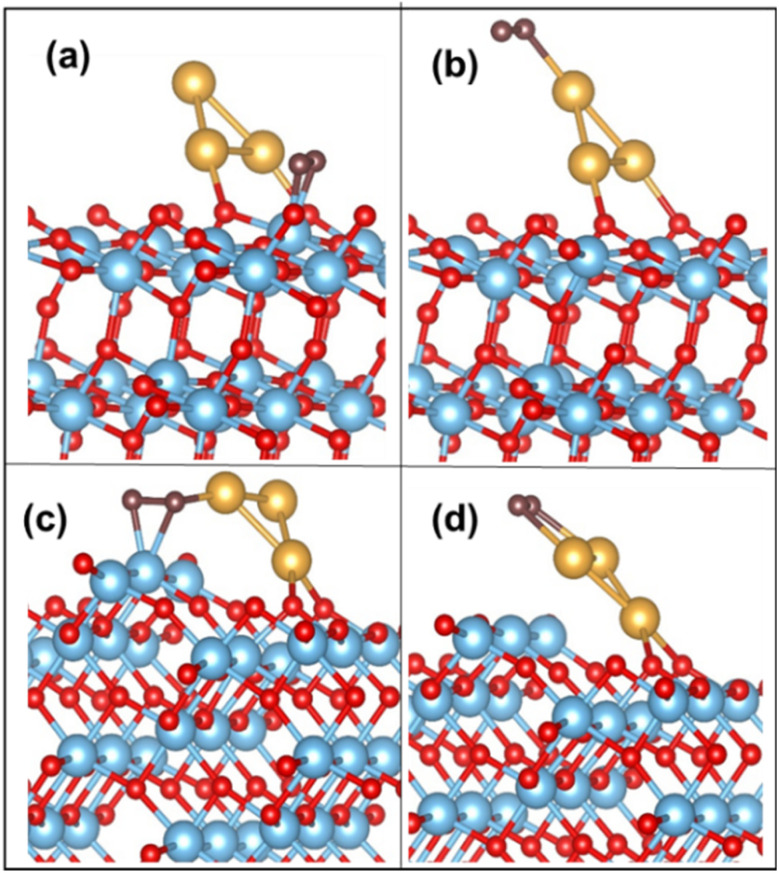
O_2_ adsorption on (a) Ti_5c_ and (b) Au on the Au_3_/TiO_2_ (101) surface; (c) Ti_4c_ and (d) Au on the Au_3_/TiO_2_ (103) surface.

On the Au_3_ supported (101) surface, the O_2_ molecule undergoes adsorption in a side-on configuration, with an adsorption energy of −0.73 eV. The O–O bond length is elongated to 1.32 Å from the gas-phase bond length of 1.23 Å, and the Ti–O bond length is 2.05 Å. The optimized configurations of O_2_ on Au_3_/TiO_2_ (101) and (103) are depicted in Fig. 4(a) and (b). This trend is similarly observed for Au_4_ and Au_5_, where O_2_ adsorbs on the Ti_5c_ site with the elongation of the O–O bond (see Table 4). The corresponding configurations are not displayed here, for the sake of brevity. In all cases, the O–O bond exhibits an average length of 1.32 Å, which puts it in the superoxide regime. The adsorbed O_2_ has a magnetic moment of 1 *μ*_B_, which indeed confirms the superoxide state.^9^

**Fig. 5:**
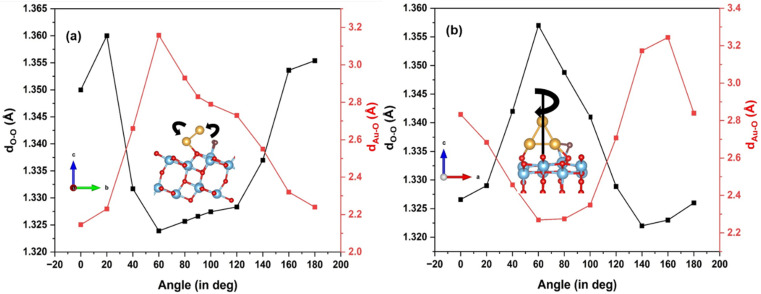
Effect of Au_3_ clusters supported on TiO_2_ rotated along (a) *a*-axis; and (b) *c*-axis, on the O–O and Au–O bond lengths.

To explore further the interaction of the adsorbed O_2_ with the Au_3_ cluster, we first rotate the cluster from 0° to 180° along the *a*-axis, as illustrated in Fig. 5(a), on the (101) surface. As the cluster is rotated through discrete angles, the O–O bond length shrinks and goes through a minimum. Correspondingly, the Au–O distance undergoes a maximum. It is interesting to note that the maximal stretching of the O–O bond occurs at angles where the cluster Au atoms are closest to the adsorbed oxygen. From the plot in Fig. 5(a), these angles are 20° and 160° with the *a*-axis, and the O–O bond stretches by 0.04 Å, with an Au–O bond distance of 2.20 Å. For an angle of 60°, where the Au_3_ cluster is farthest away from the O_2_, the O–O stretching is minimized.

We also rotate the Au_3_ cluster with respect to the *c*-axis (see Fig. 5(b)). The variation of the O–O bond length as a function of Au–O is plotted in Fig. 5(b). The O–O bonding exhibits maximum elongation at an angle of 60°, with the O–O bond length at 1.36 Å. At this point, the Au–O distance is 2.30 Å. The minimal stretching occurs at angles of 0° and 140°. The O–O bond undergoes maximum stretching when Au is closest to the O atom, forming a Ti–(O–O)–Au configuration. Thus, we understand from the above exercise that in terms of the O–O stretching, O_2_ activation may be achieved by re-orienting the Au_3_ cluster with respect to the adsorbed molecule, such that in some configurations, the Au atoms are close to the O_2_ molecule.

The above discussed rotation of the Au_3_ cluster (or Au_*n*_ cluster) may be achieved through the application of a static electric field, lattice strain or steric effects due to bulky ligands.^66,67^ There is yet another simpler way to achieve this bending, using naturally occurring surface imperfections. A stepped surface may naturally tilt the adsorbed cluster in a manner mimicking the *a*-axis rotation of Fig. 5(a). Thus, we propose that surface morphology may be used to tune cluster orientation. If we look at Au_*n*_ cluster adsorption on a (103) TiO_2_ surface, the cluster inclines itself with the “kinks” on the (103) surface. The O_2_ molecule adsorbs on the Ti_4c_ site, with Au_*n*_ at the perimeter, forming a Ti_4c_–(O–O)–Au bond. The calculated adsorption energy of O_2_ for the Au_3_ supported (103) surface is −1.66 eV, with the O–O distance stretched to 1.43 Å, corresponding to the peroxide bond length. The Au–O bond distance (*i.e.* O of O_2_) is measured at 2.07 Å. On the surface, we find that the Ti_4c_ site is slightly lifted upward, by 0.20 Å, indicating a strong Ti–O interaction. The O–O activation also occurs on the Au cluster site exhibiting an adsorption energy of −0.78 eV. These optimized configurations of O_2_ adsorbed on Au_3_/TiO_2_ (103) are shown in Fig. 4(c) and (d). These findings hold true for Au_*n*_ (*n* = 4, 5) clusters too. The corresponding binding energy values are tabulated in Table 4. The table reveals the O–O bond length in the peroxide regime. The calculated magnetic moment of the adsorbed O_2_ species is close to 0 *μ*_B_. In the case of adsorption on the low coordinated Au on the cluster itself, Au_*n*_ (*n* = 3–5) adsorb O_2_ as superoxide species, as can be seen from calculated bond lengths (compared to weak binding on the Au/(101) TiO_2_ surface) in Table 4. Thus, it appears that the interaction of the Au cluster with O_2_ has been modified by the support (103) surface, into strong chemisorption, leading to activated O_2_.

To address the question of O_2_ coverage, we calculate the adsorption energy for the second and third molecules, adsorbed on active sites on the surface. Table S1† displays the averages of adsorption energy per molecule, bond length and charge transfer (to O_2_ molecule) for both (101) and (103) surfaces. For Au_3_ supported (101), the adsorption energy of a single O_2_ molecule is −0.85 eV accompanied by an electron transfer of 0.45*e*. The second and third molecules bind within the range of physisorption. In the case of (103), the addition of a second O_2_ molecule results in an average adsorption energy of −1.10 eV, with a bond length of 1.31 Å falling within the superoxide range, and a charge transfer of 0.52*e*. The third O_2_ molecule does not undergo chemisorption and exhibits an average adsorption energy of −0.83 eV. Consequently, the Au_3_ supported (103) surface shows an optimal O_2_ binding compared to the (101) surface. The average adsorption energy per molecule for (103) is twice that of the (101) surface. Thus Au_3_/TiO_2_ (103) can adsorb more than one O_2_ molecule, in which two of them may be described as activated.

### 3.4 Stability and electronic structure considerations

To investigate the stability of the cluster configuration, molecular dynamics (MD) simulation for Au_3_ on (101) and (103) is performed. The temperature is maintained at 300 K by using the Nose thermostat^68^ for a duration of 6000 fs. Fig. S6(a)† depicts the time evolution for Au_3_/(101), along with the cluster configuration at 300 K. Monitoring the distance between Au and two-O_2c_ (*d*_1_ and *d*_2_) of TiO_2_ reveals no observable fluctuations, with an average distance ranging from 2.02 to 2.39 Å, indicating dynamic stability. A similar situation (Fig. S6(b)†) is observed for (103), with an average distance of 2.05–2.57 Å. Thus, these structures are stable at room temperature.

**Fig. 6:**
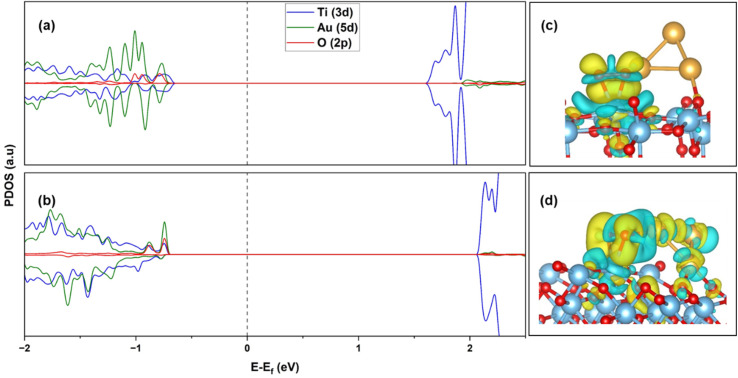
PDOS of (a) Au_3_/TiO_2_ (101), (b) Au_3_/TiO_2_ (103), after O_2_ adsorption; difference charge density plot of (c) O_2_/Au_3_/TiO_2_ (101), (d) O_2_/Au_3_/TiO_2_ (103). Yellow: charge accumulation; blue: charge depletion. Iso-surface level = 2 × 10^−3^ e Å^−3^.

The electronic structures of O_2_ adsorbed Au_3_ supported (101) and (103) TiO_2_ are compared in Fig. 6(a) and (b). For the (101) surface we see localized states of Au 5d, below the Fermi energy, as well as O 2p from the adsorbed molecule. Additionally, we also notice Ti 3d states at the edge of the valence band. All these states are broadened out a little, indicating a weakly chemisorbed state for O_2_ on the Au/TiO_2_ (101) surface. In Fig. 6(b), over the (103) surface, we see a stronger hybridization between Au 5d, Ti 3d and O 2p states, whereby the cluster states now have a more delocalized character. Au 5d states have a larger contribution at the edge of the valence band, leading to the strengthening of O_2_ adsorption. Looking at the difference charge density (or deformation density) plots in Fig. 6(c) and (d), it is clearly seen that there is no electron density accumulation in the O_2_–Au for Au/TiO_2_ (101), while for Au/TiO_2_ (103), excess charge density is present on the Au cluster, leading to enhanced O_2_ adsorption. The charge accumulation on the O_2_ is also enhanced, leading to the stretching of the O–O bond.

Calculating Bader charges in the ground state, it is observed that a total charge of 0.45*e* is transferred to the 2π* orbital of the O_2_ molecule in the Au_3_/TiO_2_ (101) configuration. This transfer can be partially from Ti on the surface, and also from Au_3_, through the surface (contributed during the relaxation of Au_3_ on the surface). With the Au–O distance of 3.70 Å, the probability of direct transfer between the Au cluster and O_2_ is very small. However, in the case of the Au_3_/TiO_2_ (103), a transfer of 0.78*e* is calculated to the O_2_ molecule. Now, the Au–O distance is 2.07 Å, and the cluster interacts directly with the O_2_ molecule. Thus, charge transfer to O_2_ is now enabled through an additional channel: transfer from Au_3_ directly. The elongation of the O–O bond length can be attributed to the stronger interaction of the Au cluster with the O_2_ molecule, enabled by the (103) step. We may also say that O_2_ is stabilized at the Au–Ti^4+^ interface through the formation of a di-σ bond involving Au–(O–O)–Ti, which is consistent with interpretations from previous work.^24,69^

**Fig. 7:**
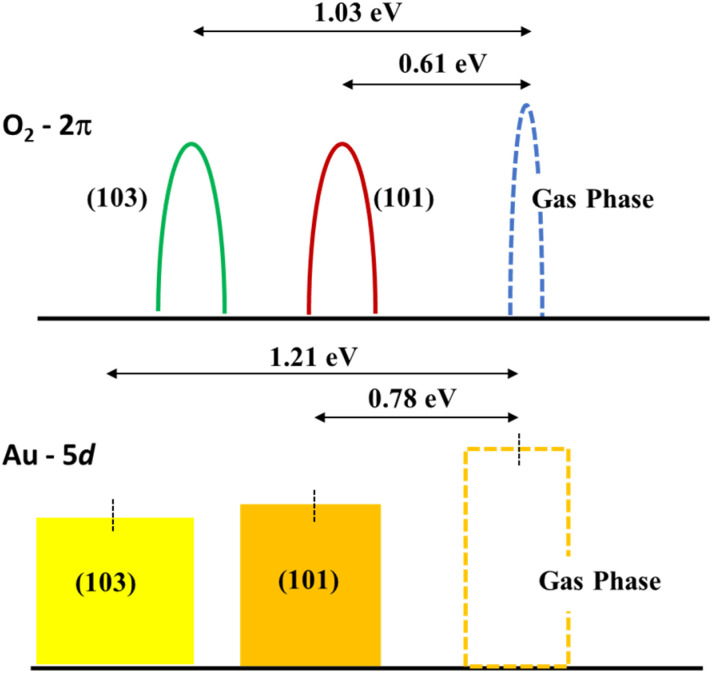
Schematic illustration of the energy level shift for (101) and (103) Au_3_/TiO_2_.

The role played by the (103) stepped surface can be understood in terms of the relative shift of the 2π energy level of the O_2_ molecule and the d-band center^70^ of Au_3_. To obtain the energy of the 2π state, we start with the ground state configuration of (101) with the Au_3_ cluster and O_2_ molecule, remove the Au_3_ completely, and then obtain the energy for the 2π state of O_2_ through the energy calculations with the molecule placed 2.10 Å and 8.82 Å above (101). The difference in these energy values is a measure of the stabilization of the 2π state due to bonding with the surface. This calculation is repeated for the (103) surface. We find that the energy of the 2π level of the O_2_ molecule decreased by 0.61 eV for (101) and 1.03 eV for (103). In a similar manner, the d-band centre for Au_3_ over both (101) and (103) was calculated without the O_2_ molecule, with Au_3_ positioned 2.06 Å and 10.70 Å above TiO_2_. The d-band centre is lowered to 0.78 eV for (101) and 1.21 eV for (103). The shifts of these two energy levels are indicated in Fig. 7. These two effects, namely the shifts of the O_2_ 2π state and the d-band centre are indicators of O_2_ activation which is enabled by (103) surface.

### 3.5 Activation barrier for O_2_ dissociation for (101) and (103)

**Fig. 8:**
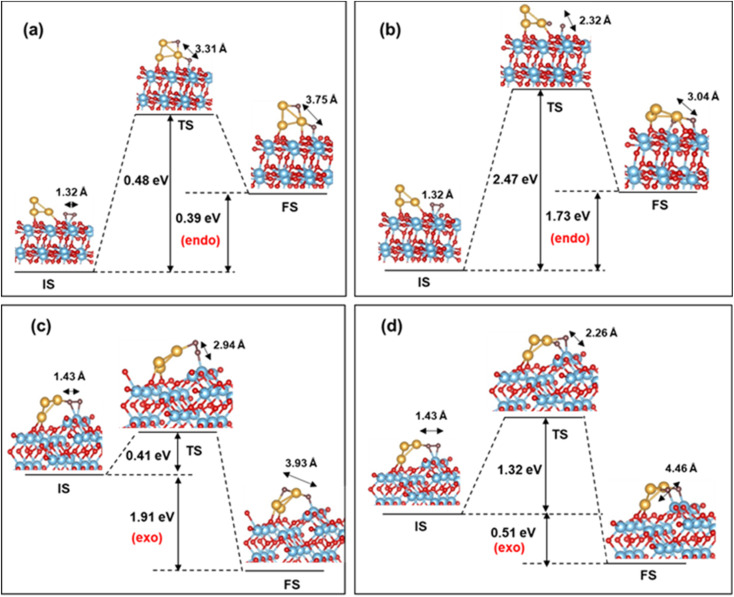
O_2_ dissociation barrier for (a) uplayered and (b) interfacial Au_3_/TiO_2_ (101); (c) uplayered and (d) interfacial Au_3_/TiO_2_ (103) (adsorbed O_2_ is in brown).

Next, we turn our attention to the dissociation process of O_2_ on (101) and (103) Au_3_/TiO_2_. An estimate of the dissociation barrier is important information, in the context of CO oxidation as well as the ORR. For the CI-NEB pathway, we take the initial state (IS) as the ground state configuration of O_2_ adsorbed on (101) and (103) Au_3_/TiO_2_. During the dissociation of O_2_, the dissociated O atom can migrate to two configurations: the “uplayered” and the “interfacial”. In the uplayered state, the oxygen atom after dissociation, attaches to the low coordinated site of Au_3_. In the interfacial state, the oxygen atom attaches itself to the neighbouring Ti. These configurations are illustrated in Fig. 8.

In the case of (101), the barrier for O_2_ dissociation at the uplayered Au is 0.48 eV, which is favourable compared to the interfacial site with a barrier of 2.47 eV (refer to Fig. 8(a) and (b)). On the other hand, for (103) the barrier (0.41 eV) is notably lower for the uplayered site, making it kinetically favourable. The barrier for the interfacial site on the (103) surface is reduced by 1.15 eV in comparison to the (101) site, and the reaction is exothermic. Consequently, (103) Au_3_/TiO_2_ exhibits superior O_2_ dissociation compared to (101) Au_3_/TiO_2_. This conclusion is in line with the fact that the former activates adsorbed O_2_ better than the latter. In contrast, the (101) or (103) TiO_2_ without Au cluster has practically no stretching of the O–O bond, and hence very small probability for O_2_ dissociation. As for non-supported Au clusters, the dissociation barrier is greater than 2 eV, according to the previous DFT studies.^24^ Thus, we predict a lowering of the O_2_ dissociation barrier in the case of Au supported stepped TiO_2_ surfaces. This is comparable to Pt (111), where the dissociation barrier is 0.45 eV, as computed by DFT.^71^ Our results are also comparable with studies on larger sized Au clusters on rutile TiO_2_ (110), where barriers of the order of 0.4–0.6 eV are observed.^72^ In particular, for the ORR, the nature of the activated state can provide information about the pathway, *i.e.* whether a four-electron or a two-electron pathway is likely to be followed, and thus the overall efficiency of the process. This is also true for any oxidation process on the surface, which involves the scission of the O–O bond in an activated state.

## 4. Conclusions

We have studied the adsorption and activation of oxygen molecules on plane (101), stepped (103) and reduced anatase TiO_2_ surfaces in this paper using plane wave DFT-based modeling. While the (101) and (103) surfaces do not activate the adsorbed O_2_ molecule, the reduced surfaces of the same adsorb O_2_ strongly. If we consider multiple O_2_ adsorption, it is seen that the reduced (103) TiO_2_ surface binds more than one O_2_ strongly. Hence, adsorption over vacancies is advantageous in terms of greater coverage of activated O_2_ when compared to (101) or (103) surfaces. Small sized Au clusters supported on the TiO_2_ surface lead to strong adsorption and activation of O_2_. In particular, the orientation of these Au clusters can tune the activation of O_2_, which depends on the proximity of Au atoms to the molecule. One can manipulate the orientation of Au clusters on TiO_2_ by adsorbing these on a step-edge of the surface. In such a configuration, we find a large stretching of the molecular O–O bond, into the peroxide regime, and strong binding. The electronic structure of the cluster–TiO_2_ interface shows good hybridization between the Au d and O p states and considerable charge transfer to the O_2_ molecule. In such an activated state, we find that the barrier for O_2_ dissociation is lower compared to the case of free Au clusters or Au clusters supported on (101) TiO_2_. Thus, we have demonstrated a simple way to utilize the morphology of the surface to suitably orient the Au cluster for enhanced O_2_ activation. Additionally, activated O_2_ may also be contributed by the active sites on the gold cluster itself. Thus, there is an overall enhancement of O_2_ activation through the Au cluster loading of the TiO_2_ surface. Experimental verification of our proposal would open a new way to realize an active catalyst surface, supported by an inexpensive and earth-abundant material and realized at low Au loading. This has important implications for various catalytic oxidation reactions.

## Data availability

The data supporting this article have been included as part of the ESI.†

## Author contributions

M. F. P.: conceptualization, formal analysis, investigation, visualization, writing – original draft. J. E. S.: conceptualization, formal analysis, investigation. R. C.: conceptualization, formal analysis, writing – review and editing, supervision, project administration.

## Conflicts of interest

There are no conflicts for the authors to declare.

## Supplementary Material

NA-OLF-D4NA00744A-s001

## References

[cit1] Stampfl C., Soon A., Piccinin S., Shi H., Zhang H. (2008). J. Phys.: Condens. Matter.

[cit2] Song J., Wang L., Zibart A., Koch C. (2012). Metals.

[cit3] Jia C., Wang X., Zhong W., Wang Z., Prezhdo O. V., Luo Y., Jiang J. (2019). ACS Appl. Mater. Interfaces.

[cit4] Wells Jr D. H., Delgass Jr W. N., Thomson K. T. (2004). J. Am. Chem. Soc..

[cit5] Zhang W., Chang J., Yang Y. (2023). SusMat.

[cit6] Costa D., Ribeiro T., Mercuri F., Pacchioni G., Marcus P. (2014). Adv. Mater. Interfaces.

[cit7] Joshi A. M., Delgass W. N., Thomson K. T. (2006). J. Phys. Chem. B.

[cit8] Allegretti F., O'Brien S., Polcik M., Sayago D. I., Woodruff D. P. (2005). Phys. Rev. Lett..

[cit9] Montemore M. M., Van Spronsen M. A., Madix R. J., Friend C. M. (2018). Chem. Rev..

[cit10] Wang Y., Ding X., Wang F., Li J., Song S., Zhang H. (2016). Chem. Sci..

[cit11] Yuan C., Wu H. B., Xie Y., Lou X. W. (2014). Angew. Chem., Int. Ed..

[cit12] Das C., Sinha N., Roy P. (2022). Small.

[cit13] Xiong W., Yin H., Wu T., Li H. (2023). Chem.–Eur. J..

[cit14] Linsebigler A. L., Lu G., Yates Jr J. T. (1995). Chem. Rev..

[cit15] Hanaor D. A. H., Sorrell C. C. (2011). J. Mater. Sci..

[cit16] Aschauer U., Chen J., Selloni A. (2010). Phys. Chem. Chem. Phys..

[cit17] Henderson M. A., Shen M., Wang Z.-T., Lyubinetsky I. (2013). J. Phys. Chem. C.

[cit18] Thompson T. L., Yates J. T. (2006). Chem. Rev..

[cit19] Pang C. L., Lindsay R., Thornton G. (2013). Chem. Rev..

[cit20] Puthiyaparambath M. F., Chatanathodi R. (2023). Phys. Rev. Mater..

[cit21] Hemmingson S. L., Campbell C. T. (2017). ACS Nano.

[cit22] Li X., Yang X., Huang Y., Zhang T., Liu B. (2019). Adv. Mater..

[cit23] Pan C.-J., Tsai M.-C., Su W.-N., Rick J., Akalework N. G., Agegnehu A. K., Cheng S.-Y., Hwang B.-J. (2017). J. Taiwan Inst. Chem. Eng..

[cit24] Liu Z.-P., Gong X.-Q., Kohanoff J., Sanchez C., Hu P. (2003). Phys. Rev. Lett..

[cit25] Haruta M. (1997). Catal. Today.

[cit26] van Bokhoven J. A., Louis C., Miller J. T., Tromp M., Safonova O. V., Glatzel P. (2006). Angew. Chem., Int. Ed..

[cit27] Weiher N., Beesley A. M., Tsapatsaris N., Delannoy L., Louis C., van Bokhoven J. A., Schroeder S. L. M. (2007). J. Am. Chem. Soc..

[cit28] Hayashi T., Tanaka K., Haruta M. (1998). J. Catal..

[cit29] Akita T., Tanaka K., Tsubota S., Haruta M. (2000). J. Electron Microsc..

[cit30] Zhang Y., Liu J.-X., Qian K., Jia A., Li D., Shi L., Hu J., Zhu J., Huang W. (2021). Angew. Chem., Int. Ed..

[cit31] Green I. X., Tang W., Neurock M., Yates J. T. (2014). Acc. Chem. Res..

[cit32] Siemer N., Lüken A., Zalibera M., Frenzel J., Muñoz-Santiburcio D., Savitsky A., Lubitz W., Muhler M., Marx D., Strunk J. (2018). J. Am. Chem. Soc..

[cit33] Remediakis I. N., Lopez N., Nørskov J. K. (2005). Appl. Catal., A.

[cit34] Valden M., Lai X., Goodman D. W. (1998). Science.

[cit35] Wan W., Nie X., Janik M. J., Song C., Guo X. (2018). J. Phys. Chem. C.

[cit36] Tada K., Koga H., Hayashi A., Kondo Y., Kawakami T., Yamanaka S., Okumura M. (2017). Bull. Chem. Soc. Jpn..

[cit37] Lakshmikanth K. G., Puthiyaparambath M. F., Chatanathodi R. (2022). Surf. Sci..

[cit38] Petitto S. C., Marsh E. M., Langell M. A. (2006). J. Phys. Chem. B.

[cit39] Diebold U. (2003). Surf. Sci. Rep..

[cit40] Chusuei C. C., Lai X., Luo K., Goodman D. W. (2000). Top. Catal..

[cit41] Gong X.-Q., Selloni A., Batzill M., Diebold U. (2006). Nat. Mater..

[cit42] Gong X., Selloni A. (2007). J. Catal..

[cit43] Rieboldt F., Bechstein R., Besenbacher F., Wendt S. (2014). J. Phys. Chem. C.

[cit44] Gao Y., Elder S. A. (2000). Mater. Lett..

[cit45] Onishi H., Aruga T., Egawa C., Iwasawa Y. (1988). Surf. Sci..

[cit46] Du X., Huang Y., Pan X., Han B., Su Y., Jiang Q., Li M., Tang H., Li G., Qiao B. (2020). Nat. Commun..

[cit47] Kresse G., Furthmüller J. (1996). Phys. Rev. B: Condens. Matter Mater. Phys..

[cit48] Kresse G., Hafner J. (1993). Phys. Rev. B: Condens. Matter Mater. Phys..

[cit49] Blöchl P. E. (1994). Phys. Rev. B: Condens. Matter Mater. Phys..

[cit50] Kresse G., Joubert D. (1999). Phys. Rev. B: Condens. Matter Mater. Phys..

[cit51] Perdew J. P., Burke K., Ernzerhof M. (1996). Phys. Rev. Lett..

[cit52] Grimme S. (2006). J. Comput. Chem..

[cit53] Grimme S., Antony J., Ehrlich S., Krieg H. (2010). J. Chem. Phys..

[cit54] Monkhorst H. J., Pack J. D. (1976). Phys. Rev. B: Solid State.

[cit55] Yan L., Chen H. (2014). J. Chem. Theory Comput..

[cit56] Morgan B. J., Watson G. W. (2007). Surf. Sci..

[cit57] Burdett J. K., Hughbanks T., Miller G. J., Richardson Jr J. W., Smith J. V. (1987). J. Am. Chem. Soc..

[cit58] Henkelman G., Uberuaga B. P., Jónsson H. (2000). J. Chem. Phys..

[cit59] Bader R. F. W. (1991). Chem. Rev..

[cit60] Lazzeri M., Vittadini A., Selloni A. (2001). Phys. Rev. B: Condens. Matter Mater. Phys..

[cit61] Zhou Z., Yu Y., Ding Z., Zuo M., Jing C. (2018). Eur. J. Inorg. Chem..

[cit62] Herman G. S., Sievers M. R., Gao Y. (2000). Phys. Rev. Lett..

[cit63] Peng H., Li J., Li S.-S., Xia J.-B. (2008). J. Phys.: Condens. Matter.

[cit64] Gentile A., Ruffino F., Grimaldi M. G. (2016). Nanomaterials.

[cit65] Vittadini A., Selloni A. (2002). J. Chem. Phys..

[cit66] Richter B., Kuhlenbeck H., Freund H.-J., Bagus P. S. (2004). Phys. Rev. Lett..

[cit67] Deng G., Malola S., Yuan P., Liu X., Teo B. K., Häkkinen H., Zheng N. (2021). Angew. Chem., Int. Ed..

[cit68] Nosé S. (1984). J. Chem. Phys..

[cit69] Green I. X., Tang W., Neurock M., Yates Jr J. T. (2014). Acc. Chem. Res..

[cit70] Hammer B., Nørskov J. K. (2000). Adv. Catal..

[cit71] Jennings P. C., Aleksandrov H. A., Neyman K. M., Johnston R. L. (2014). Nanoscale.

[cit72] Green I. X., Tang W., McEntee M., Neurock M., Yates Jr J. T. (2012). J. Am. Chem. Soc..

