# Supplementary material for: Tailoring surface morphology on anatase TiO2 supported Au nanoclusters: implications for O2 activation
Source: Nanoscale Adv. 2024 Sep 18;6(23):5897–908. doi: 10.1039/d4na00744a (PMC11441460; doi:10.1039/d4na00744a)
Supplement: NA-006-D4NA00744A-s001 [file NA-006-D4NA00744A-s001.pdf]

Supplementary Information (SI) for Nanoscale Advances.
This journal is © The Royal Society of Chemistry 2024

Supplementary Information

Tailoring surface morphology on anatase TiO_2 supported Au nanoclusters: implications for O_2 activation

Muhammed Fasil Puthiyaparambath, Julian Ezra Samuel, and Raghu Chatanathodi*
Department of Physics
National Institute of Technology Calicut, Calicut, Kerala, 673601, India
*Corresponding author: raghuc@nitc.ac.in

S1: Hydration energy calculations

To find out the surface energy of the hydrated surface (γ'), we used the following expression $\gamma' = \gamma + {nE}/{A}$, where γ, n, E, and A are the surface energy of the clean surface, no. of H_2O molecule, the adsorption energy of the water molecule and area of the slab respectively.

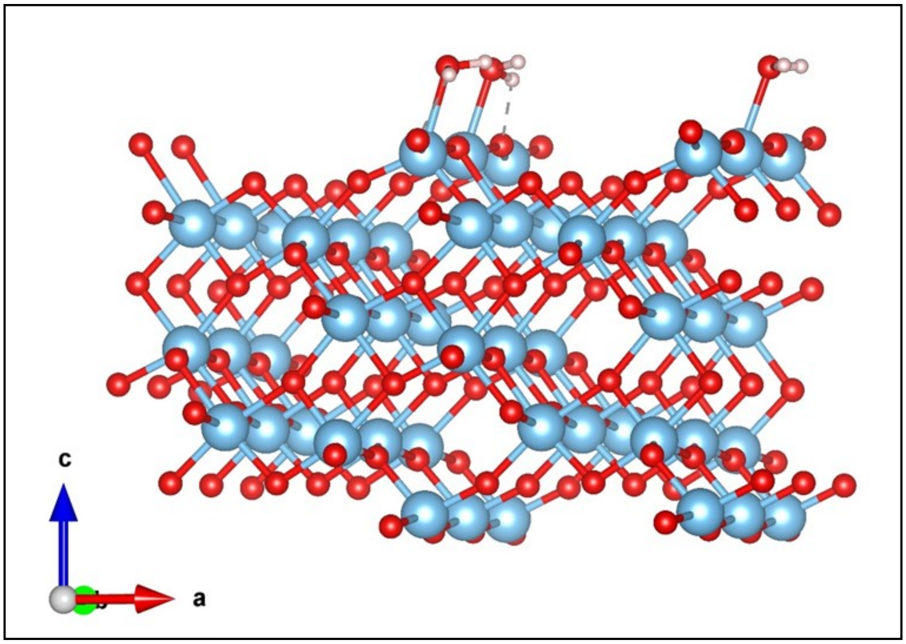

Figure S1. Optimized configuration of hydrated (103) surface

The values obtained for hydration surface energy as per the above equation for the (101) surface are 0.35, 0.49 and 0.61 J/m^2, while for the (103) surface, they are 0.67, 0.75 and 0.85 J/m^2 for the 1/6, 1/3 and 1/2 monolayer (ML) coverage, respectively. Notably, the decrease in hydration surface energies is greater for (103) compared to (101) at all values of coverage considered here, indicating that hydration stabilizes the (103) surface more effectively.

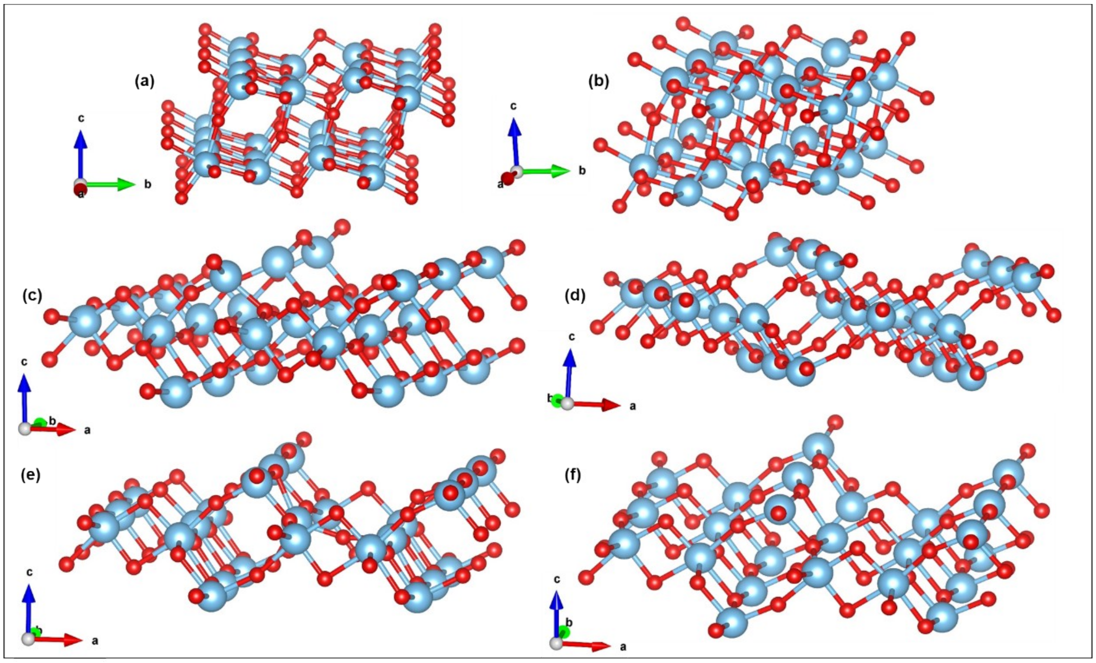

Figure S2. Optimized geometries of reduced (101) (a) V_{2c} (b) V_{3c} and (103) (c) V_{2c1} (d) V_{2c2} (e) V_{2c3} (f) V_{3c1}

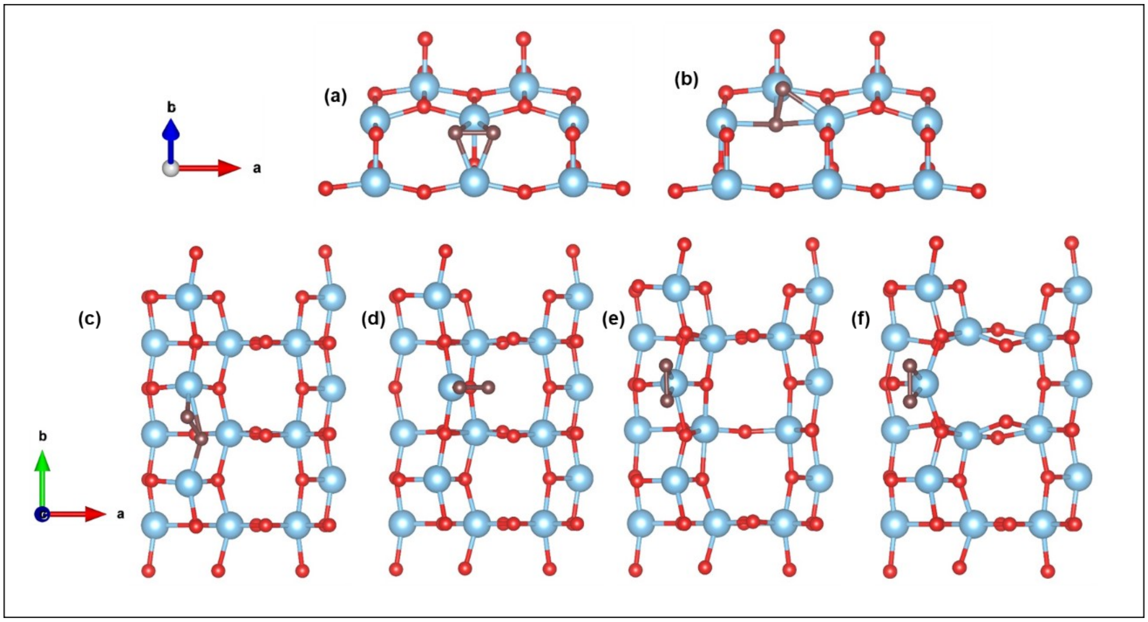

Figure S3. Optimized geometries of O_2 adsorption on reduced (101) (a) V_{2c} (b) V_{3c} and (103) (c) V_{2c1} (d) V_{2c2} (e) V_{2c3} (f) V_{3c1}

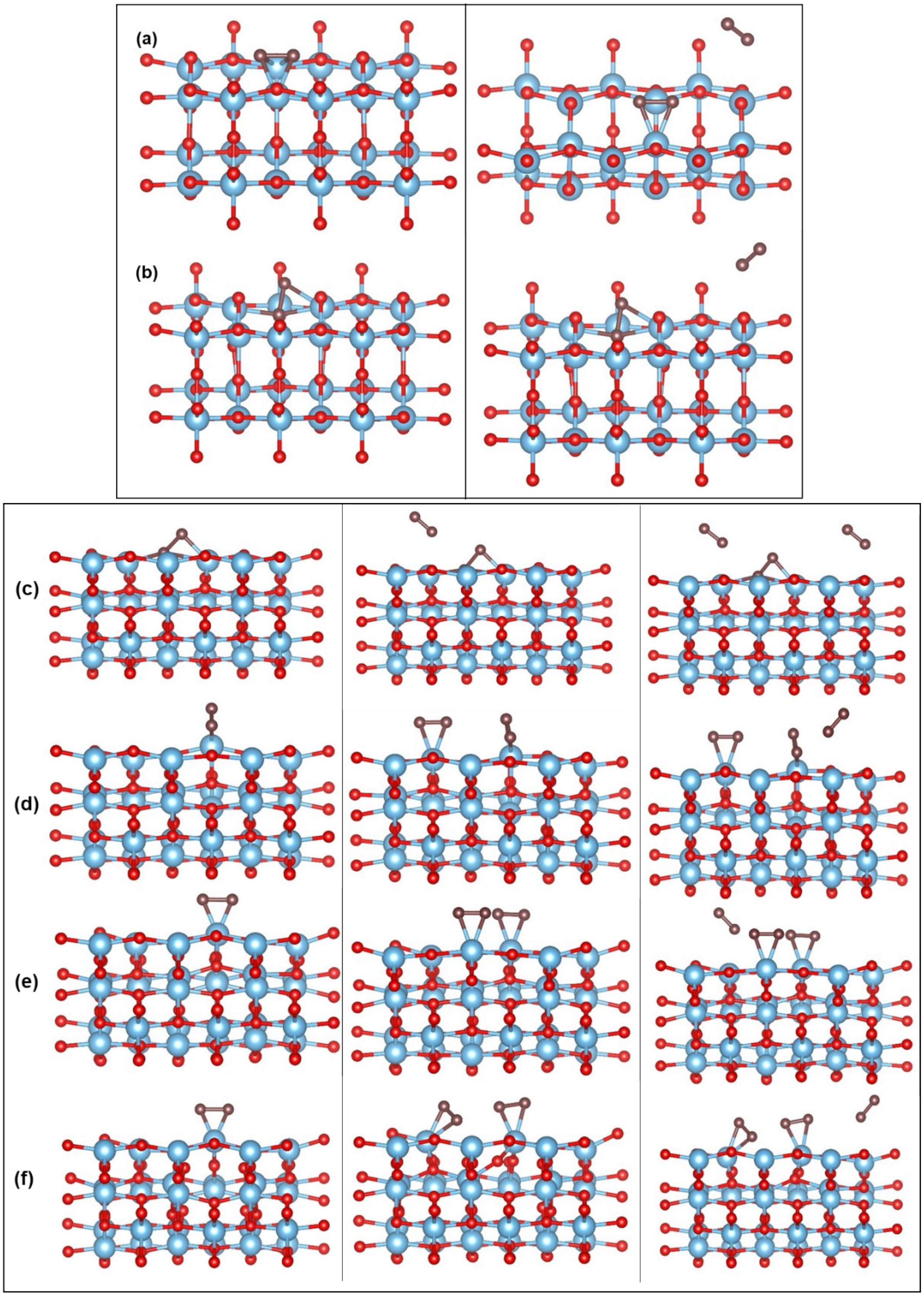

Figure S4. Coverage studies on (101) and (103) TiO_2 surfaces for (a) O_{2c} (b) O_{3c} (c) O_{2c1} (d) O_{2c2} (e) O_{2c3} (f) O_{3c1}

S2: CI-NEB calculations

We calculated the barrier using CI-NEB to determine the energy barrier for diffusion of the dissociated oxygen atom into the lattice. The barrier for V_{2c} and V_{3c} for the TiO_2 (101) surface is zero. Similarly, the V_{2c1} (103) surface also follows a barrierless pathway. However, V_{2c2}, V_{2c3} and V_{3c1} of (103) have a non-zero barrier. The initial states (IS) and final states (FS) were obtained after structural relaxation. At the transition state (TS) the O-O bond lengths are approximately 2.49 Å, 3.78 Å and 3.36 Å for V_{2c2}, V_{2c3} and V_{3c1}, respectively. These configurations have a barrier of 1.15 eV, 1.98 eV and 0.78 eV. For V_{3c1}, the reaction is exothermic, while the remaining two configurations are endothermic.

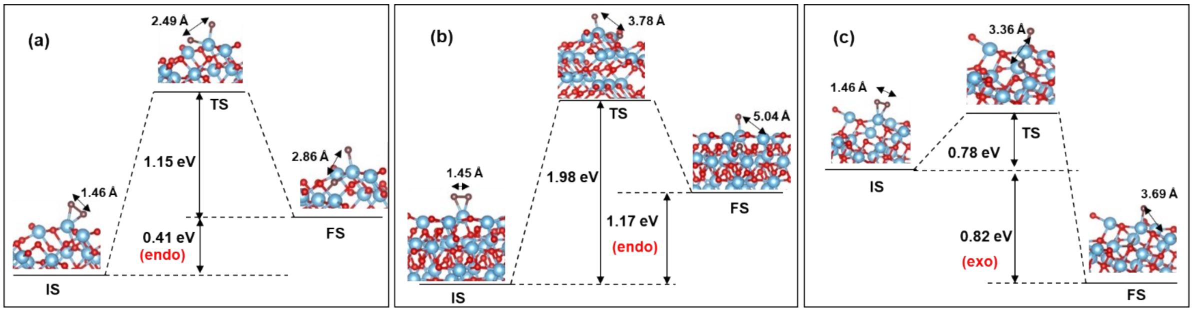

Figure S5. The diffusion barrier for lattice trapping of O_2 on (a) V_{2c2} (b) V_{2c3} (c) V_{3c1}

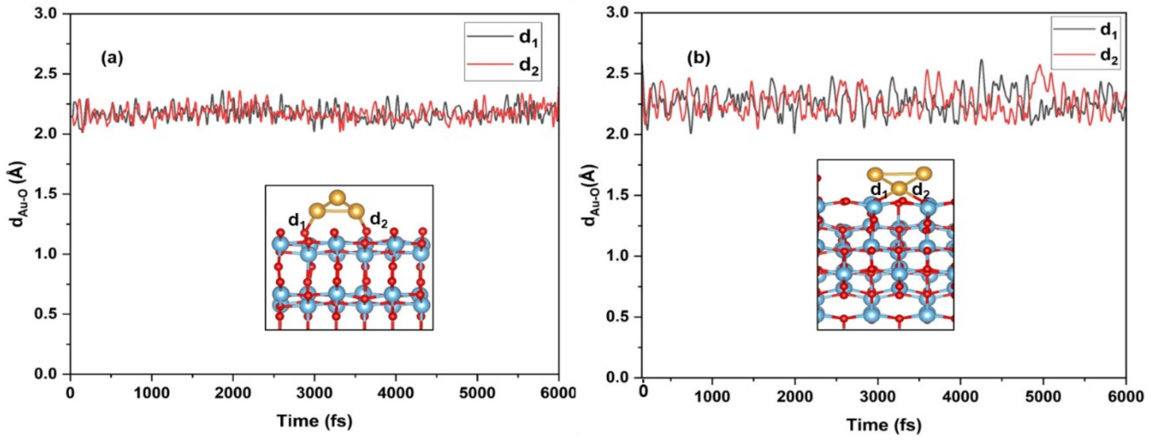

Figure S6. Time evolution of $d_{Au\text{-}O2c}$ (d_1 and d_2) for Au_3 supported on (a) (101) and (b) (103). The time trace shows the distance between Au and the two-coordinated oxygen atom of TiO_2. The time traces show that the distance Au-O_{2c} is more or less the same at 0K and 300K.

Table S1: Adsorption energy per O_2 molecule on (101) and (103) Au_3/TiO_2: a comparison.

No. of O_2 molecules	(101)			(103)		
	E_{ads} (eV)	d_{O-O} (Å)	Q |e|	E_{ads} (eV)	d_{O-O} (Å)	Q |e|
1	-0.85	1.32	0.45	-1.66	1.43	0.78
2	-0.55	1.29	0.30	-1.10	1.37	0.52
3	-0.44	1.27	0.20	-0.83	1.33	0.41
